# Personalized normative feedback interventions targeting hazardous alcohol use and alcohol-related risky sexual behavior in Swedish university students: A randomized controlled replication trial

**DOI:** 10.1016/j.abrep.2020.100300

**Published:** 2020-09-04

**Authors:** Claes Andersson

**Affiliations:** Faculty of Health and Society, Malmö University, SE205 02 Malmö, Sweden

**Keywords:** Personalized feedback intervention, Young adults, Alcohol, Sexual risk taking, AQW, Actual Quantity Week, AFW, Actual Frequency Week, AQO, Actual Quantity Occasion, AFDPS, Actual Frequency of Drinking Prior Sex, AQDPS, Actual Quantity of Drinking Prior Sex, ARNEG, Alcohol-related Negative Consequences, ARSEX, Alcohol-related Sexual Consequences

## Abstract

**Introduction:**

This study replicates two US intervention studies using personalized normative feedback (PNF) on alcohol-related risky sexual behavior (RSB).

**Methods:**

In a randomized controlled trial, 654 Swedish university students were assigned to an alcohol only intervention, an alcohol-related RSB only intervention, a combined alcohol and alcohol-related RSB intervention, an integrated alcohol and alcohol-related RSB intervention, or control. Follow-up assessments were made at 3 and 6 months post-intervention.

**Results:**

In comparison to controls, drinks per week were reduced at 3 months in the Alcohol Only, Combined, and Integrated intervention groups. Frequency and quantity of drinking before sex were reduced at 3- and 6-month follow-up for the Sex Only, Combined, and Integrated intervention groups. The Alcohol Only intervention showed significant results on frequency of drinking before sex at 3 months, and on quantity of drinking before sex at 6 months. The Combined intervention had reduced outcomes on alcohol-related consequences and on alcohol-related sexual consequences at both follow-ups. Alcohol Only and Integrated interventions showed effects on both outcomes regarding consequences at 6 months, and the Sex Only group showed effects on sexual consequences at 6 months.

**Conclusions:**

It is concluded that PNF interventions offer considerable positive effects, and could be used to reduce alcohol-related RSB in Swedish university students.

## Highlights

• Effects of normative feedback interventions on alcohol-related sexual behavior is studied.
• By using a factorial design, several positive effects could be identified.
• It is concluded that normative feedback interventions reduce alcohol-related sexual behavior.

## 1 Introduction

Hazardous drinking and heavy episodic drinking have been associated with risky sexual behavior (RSB), which is defined as a wide range of behaviors associated with increased risk of a variety of negative consequences relating to sex, such as contracting or transmitting disease or the occurrence of unwanted pregnancy (Cooper, 2006, Rehm et al., 2012). These problem behaviors are frequent in young adults (Kerr et al., 2009, Lyons et al., 2014), an age group where approximately half the population are university students (OECD, 2018). A recent review reports only a few intervention studies on alcohol-related RSB, and even fewer studies on young adults at university (Ahankari, Wray, Jomeen, & Hayer, 2019). None of these studies were conducted in Sweden.

Studies conducted in Sweden (Andersson, 2015), and elsewhere (Dotson et al., 2015, Lewis and Neighbors, 2006), have shown that personalized normative feedback (PNF) interventions can be used to reduce hazardous drinking in young adults at university. These interventions are based on the theory of normative perception, meaning that an individual’s behavior is shaped by often selective judgments or misperceptions about the behavior of others (Bandura, 1977, Bandura, 1986). Interventions are designed to correct misperceptions regarding the prevalence of problematic behavior, by showing individuals engaging in such behaviors that their own behavior differs from actual norms (Lewis & Neighbors, 2006). The feedback is often followed by useful recommendations on how to change the problem behavior.

PNF have been used in two consecutive studies by Lewis and colleagues (2014; 2019) targeting hazardous drinking and alcohol-related RSB in college students in the US. In both studies, alcohol-related RSB is limited to engaging in sexual behavior with multiple or casual partners under the influence of alcohol.

In the first study (Lewis et al., 2014), participants were randomized to an alcohol-only intervention, or to an alcohol-related RSB-only intervention, a combined alcohol and alcohol-related RSB intervention, or to a control group. Follow-ups were made 3 and 6 months post-intervention. For the alcohol-only group, frequency and quantity of alcohol use were reduced at both follow-ups in comparison to the control group. For the sex-only group, frequency of drinking before sex was reduced at the 3-month follow-up compared with controls. For the combined group, frequency and quantity of alcohol use, as well as frequency of drinking before sex, were reduced at 3 months compared with the control group.

In the second study (Lewis et al., 2019), participants were randomized to either the same combined intervention used in the preceding study, or to an integrated intervention where all PNF-content referred to situations where alcohol was consumed in conjunction with sex, or to a control group. In contrast to the additive approach, normative comparisons in the integrated intervention focuses only on intoxication as a barrier to risk reduction in sexual situations, meaning that only one set of information needs to be understood and recalled. Follow-ups were made 1 and 6 months post-intervention. At the first follow-up, frequency of drinking before sex was reduced in both intervention groups compared to the control group. In the combined group, quantity of alcohol consumed was lower, and in the integrated group alcohol-related negative consequences were reduced, both results in comparison to the control group and at the 1-month post-intervention assessment.

### 1.1 The present study

The purpose of the present study was to replicate the two previous personalized normative intervention studies in one single intervention study in Swedish university students. The present study includes 3- and 6-month post-intervention assessments in the following five groups: Alcohol Only, Sex Only, Combined, Integrated, and Control. Based on previous findings, it was expected that the Alcohol Only group would show reductions in alcohol outcomes, that the Sex Only group would show reductions on alcohol-related RSB outcomes, and that the Combined and Integrated groups would show similar reductions in both alcohol outcomes and alcohol-related RSB outcomes, relative to the control group.

## 2 Methods

### 2.1 Participants and procedures

**Fig. 1:**
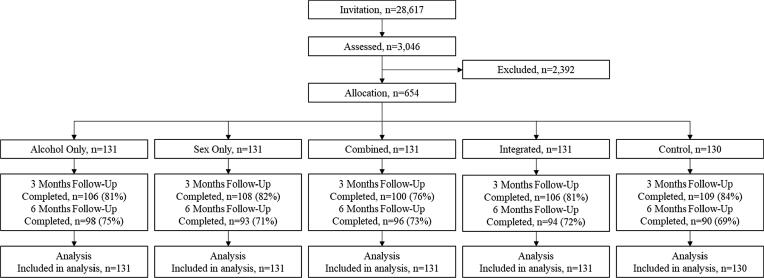
Participant flow through the study process.

Participant flow through this study is presented in Fig. 1.

A total of 28,617 students from six universities in Sweden were selected from university records. Selection criteria were 30 years or younger and studying at least half time. Selected students were sent one invitation by email for participation in a sex and alcohol intervention study. The email was opened by 13,869 students, 3046 of whom submitted informed consent and complete responses to an online screening survey comprising 229 questions.

A total of 654 students met eligibility criteria and constituted the final sample. Eligible participants reported (a) age 18–30 years, (b) heterosexual, (c) sexually active in the previous 3 months, (d) scores on the Alcohol Use Identification Test (AUDIT; Saunders et al., 1993, Reinert and Allen, 2002) indicating hazardous alcohol use (≥6 for women/≥8 men), and (e) having at least four drinks (for women) or five drinks (for men) on one occasion in the previous three months, indicating heavy episodic drinking (National Institute on Alcohol Abuse and Alcoholism, 2007).

Eligible participants, 62% female and mean age 23.7 (SD 2.7), were randomized into one of five groups using stratified random assignment based on gender and age group (18–25, 26–30). Random assignment was administered automatically using a computer algorithm, generating blocks of five to keep cell sizes equal.

Randomized students were informed by email that they had been selected for the intervention. The content could be viewed digitally by entering the same PIN code that participants had selected when giving consent to participation. Access to intervention content was not limited in time.

The intervention included descriptive normative comparisons based on the total sample of respondents at the initial screening assessment. Comparisons were presented in text and bar graph format, and included information about the student’s own behavior, their perceptions of typical behavior in their sex and age group, and typical actual behavior in their sex and age group.

Follow-ups were made after 3 and 6 months. At both follow-ups, the initial invitation was sent by email. Non-respondents were reminded by email up to four times, by text messages up to two times, and by one phone call. Of the 654, 529 (81%) and 479 (72%) completed the 3-month and 6-month follow-up assessments, respectively.

At all assessments, and as an incentive for participation, respondents were included in a lottery arranged by the non-profit charitable fund Save the Children. At the initial screening, prizes in the lottery included 33 gift vouchers valued between SEK 500–10,000 (approximately USD 50–1000). At follow-ups, prizes included 80 gift vouchers each valued at SEK 500 (approx. USD 50).

### 2.2 Interventions

#### 2.2.1 Alcohol Only PNF (Alcohol Only)

Normative comparisons of (a) frequency of drinking per week, (b) quantity of drinking per week, and (c) quantity of drinking per occasion. Feedback was given on individual expectancies and consequences in relation to alcohol use. Tips were given on useful protective strategies in relation to alcohol use.

#### 2.2.2 Alcohol-related Risky Sexual Behavior Only PNF (Sex Only)

Normative comparisons on (a) quantity of sexual partners, (b) frequency of sex with a casual partner, (c) frequency of drinking in conjunction with sex, and (d) quantity of drinking in conjunction with sex. Feedback was given on individual expectancies and consequences in relation to alcohol use in conjunction with sex. Tips were given on protective behavioral strategies in relation to alcohol use in conjunction with sex.

#### 2.2.3 Combined Alcohol and Alcohol-related Risky Sexual Behavior PNF (Combined)

Normative comparisons offered to the Alcohol Only group (see above), followed by the same comparisons offered to the Sex Only group (see above). Feedback was given on individual expectancies and consequences in relation to alcohol use and alcohol use in conjunction with sex. Tips were given on protective behavioral strategies in relation to alcohol use and alcohol use in conjunction with sex.

#### 2.2.4 Integrated Alcohol and Alcohol-related Risky Sexual Behavior PNF (Integrated)

Normative comparisons on (a) quantity of sexual partners when under the influence of alcohol, (b) quantity of casual sex partners when under the influence of alcohol, (c) frequency of drinking in conjunction with sex, and (d) quantity of drinking in conjunction with sex. Feedback was given on individual expectancies and consequences in relation to alcohol use and alcohol use in conjunction with sex. Tips were given on protective behavioral strategies in relation to alcohol use and alcohol use in conjunction with sex.

#### 2.2.5 Attention Control feedback (Control)

Normative comparisons on (a) training and physical activity, and (b) diet of fish, fruit, and vegetables. Recommendations from the Swedish Food Agency were given on physical activity and diet.

Table 1 summarizes intervention content by intervention group. It should be noted that normative comparisons in the combined intervention only focus on intoxication as a barrier to risk reduction in sexual situations, while the combined intervention has an additive approach.

**Table 1:** Intervention content by intervention group.

		Alcohol Only	Sex Only	Combined	Integrated
*Normative Comparisons*					
Alcohol	Frequency/week	X		X	
	Quantity/week	X		X	
	Quantity/occasion	X		X	
Sex	Quantity sexual partners		X	X	
	Frequency casual partner		X	X	
Alcohol and Sex	Frequency alcohol/sex		X	X	X
	Quantity alcohol/sex		X	X	X
	Quantity partners/alcohol				X
	Quantity casual partners/alcohol				X

*Feedback*					
Expectancies	Alcohol use	X		X	X
	Alcohol use when sex		X	X	X
Consequences	Alcohol use	X		X	X
	Alcohol use when sex		X	X	X

*Recommendations*					
Alcohol use		X		X	X
Alcohol use when sex			X	X	X

### 2.3 Measures

#### 2.3.1 Actual Quantity Week (AQW)

The Daily Drinking Questionnaire (DDQ; Collins, Parks, & Marlatt, 1985) was used to assess the number of standard drinks per week in the previous three months. Participants were asked to report the average number of standard drinks consumed on each day of a typical week. Weekly drinking was computed by totaling the number of drinks for each day of the week.

#### 2.3.2 Actual Frequency Week (AFW)

The Quantity/Frequency/Peak Alcohol Use Index (Dimeff, Baer, Kivlahan, & Marlatt, 1999) was used to assess typical frequency of drinking per week during the previous three months. Participants were asked to report how many times they had consumed alcohol per week. Response options ranged from 0 to 30 times.

#### 2.3.3 Actual Quantity Occasion (AQO)

The Quantity/Frequency/Peak Alcohol Use Index (Dimeff et al., 1999) was used to assess typical quantity of drinks per occasion during the previous three months. Participants were asked to report the typical number of drinks per drinking session. Response options ranged from 0 to 25 or above.

#### 2.3.4 Actual Frequency of Drinking Prior Sex (AFDPS)

Frequency of alcohol use in conjunction with digital, oral, vaginal, and anal sex over the previous three months was assessed by one single question originally developed by Lewis, Lee, Patrick, and Fossos (2007). Participants were asked to report how many times they had consumed alcohol before or during sexual encounters. Response options ranged from 0 to 25 times or above.

#### 2.3.5 Actual Quantity of Drinking Prior Sex (AQDPS)

Typical quantity of standard drinks consumed in conjunction with digital, oral, vaginal, and anal sex over the previous three months was assessed by one single question developed by Lewis et al. (2007). Participants were asked to report how many drinks on average they had consumed before or during sexual encounters. Response options ranged from 0 to 25 or above.

#### 2.3.6 Alcohol-related Negative Consequences (ARNEG)

The Brief Young Adult Alcohol Consequences Questionnaire (BYAACQ; Kahler, Strong, & Read, 2005) was used to assess alcohol-related negative consequences during the previous three months. Participants indicated which items on a list of 24 potential problems they experienced because of their drinking.

#### 2.3.7 Alcohol-related Sexual Consequences (ARSEX)

The Alcohol-related Sexual Consequences Scale developed by Lewis et al. (2019) was used to assess alcohol-related sexual consequences during the previous three months. Sexual behavior includes digital, oral, vaginal, and anal sex. Participants indicated which items on a list of 41 potential problems they had experienced because of drinking alcohol.

### 2.4 Analysis

The analysis aimed to ascertain whether the four active interventions offered significant reductions relative to attention control on seven outcome measures assessed at 3 and 6 months post-intervention. The same zero-adjusted mixture count models described in detail by Lewis and colleagues (2014) were used to analyze the two follow-ups in separate models. Each model includes treatment contrasts with attention control as the reference category, gender, and the baseline value of the outcomes of covariates. Rate ratios (RRs) and 95% confidence intervals (CIs) were used to interpret coefficients. Data were analyzed according to the intention-to-treat approach. All analyses were performed in the statistical software R, using packages for negative binomial regression and hurdle models, respectively.

## 3 Results

Means and standard deviations for behavior outcomes by treatment group are shown in Table 2.

**Table 2:** Means and standard deviations for behavioral outcomes by treatment condition.

		Alcohol Only	Sex Only	Combined	Integrated	Control
AQW	T0	10.15(5.21)	10.00(5.47)	10.50(6.22)	10.37(5.56)	10.12(5.85)
	T3	8.44(5.71)	9.71(6.16)	8.01(5.61)	8.65(6.11)	10.25(6.79)
	T6	9.02(6.41)	9.59(6.63)	8.45(5.75)	9.07(6.22)	9.77(7.17)

AFW	T0	2.24(1.04)	2.07(0.80)	2.21(1.00)	2.15(0.98)	2.26(1.06)
	T3	1.98(1.15)	2.02(0.93)	1.91(1.06)	1.88(1.07)	2.29(1.23)
	T6	2.09(1.15)	1.98(1.02)	1.97(1.10)	1.98(1.13)	2.22(1.30)

AQO	T0	4.78(1.68)	4.97(1.98)	4.92(1.99)	5.17(2.44)	4.69(1.93)
	T3	4.06(1.72)	4.58(2.13)	4.07(1.86)	4.41(1.87)	4.33(1.91)
	T6	4.04(1.62)	4.49(2.19)	4.20(1.72)	4.37(1.93)	4.11(1.97)

AFDPS	T0	3.70(4.37)	3.82(4.04)	3.89(3.72)	3.79(3.76)	3.81(3.16)
	T3	3.12(1.62)	2.84(2.27)	2.62(1.73)	2.63(2.94)	3.76(2.81)
	T6	3.27(2.11)	2.67(2.13)	2.66(1.93)	2.61(2.33)	3.57(2.74)

AQDPS	T0	3.85(3.50)	3.92(3.11)	3.90(2.71)	3.98(3.08)	3.86(2.63)
	T3	3.36(2.75)	3.02(2.83)	2.88(2.53)	2.94(2.44)	3.61(2.42)
	T6	3.21(2.55)	3.02(2.72)	2.89(2.58)	2.95(2.17)	3.49(2.49)

ARNEGC	T0	8.79(3.90)	8.80(4.09)	8.60(3.86)	8.83(4.12)	8.68(4.53)
	T3	7.64(4.86)	8.06(4.15)	6.90(4.86)	7.85(4.80)	8.53(4.19)
	T6	7.05(4.84)	7.57(4.48)	6.55(4.45)	7.10(4.25)	8.44(4.76)

ARSEXC	T0	7.80(5.28)	7.75(5.23)	7.58(5.12)	7.55(5.05)	7.65(5.21)
	T3	5.15(4.99)	5.55(5.06)	5.21(4.79)	5.78(6.15)	7.17(5.60)
	T6	5.35(5.20)	5.67(5.22)	4.96(4.54)	5.10(4.66)	7.19(6.31)

Note. AQW = Actual Quantity Week; AFW = Actual Frequency Week; AQO = Actual Quantity Occasion; AFDPS = Actual Frequency of Drinking Prior Sex; AQDPS = Actual Quantity of Drinking Prior Sex; ARNEG = Alcohol-related Negative Consequences; ARSEX = Alcohol-related Sexual Consequences; T = Time.

**Fig. 2:**
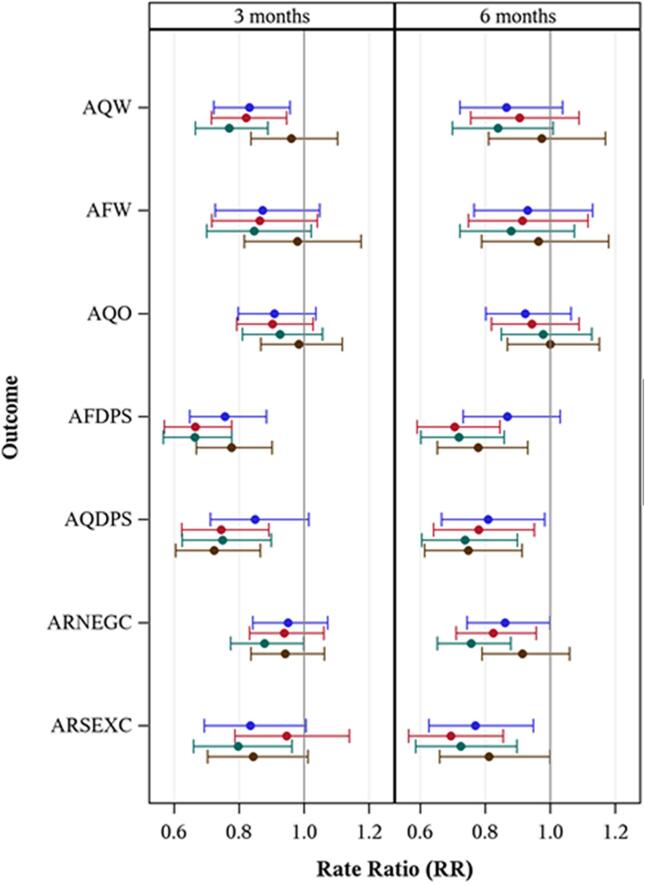
Rate Ratios (RR) and 95% confidence intervals (CIs) for RRs comparing treatment conditions to control at 3 and 6 months post-intervention outcomes. Note:  = Alcohol Only,  = Integrated,  = Combined,  = Sex Only. AQW = Actual Quantity Week; AFW = Actual Frequency Week; AQO = Actual Quantity Occasion; AFDPS = Actual Frequency of Drinking Prior Sex; AQDPS = Actual Quantity of Drinking Prior Sex; ARNEG = Alcohol-related Negative Consequences; ARSEX = Alcohol-related Sexual Consequences. (For interpretation of the references to colour in this figure legend, the reader is referred to the web version of this article.)

Fig. 2 shows rate ratios (RRs) and 95% confidence intervals (CIs) for the four active treatments relative to control, for each of the seven outcomes at 3 months and 6 months, controlling for gender and baseline outcome behavior. CIs that do not exceed 1 are significant at the p < .05 level.

The upper section presents results on three measures of alcohol use. For drinks per week (AQW), a significant decrease was found at 3-month follow-up for three intervention groups: Alcohol Only, Combined, Integrated.

The middle section presents results on two measures of alcohol use in conjunction with sex. On both measures, significant effects could be seen at both 3- and 6-month follow-up for three intervention groups: Sex Only, Combined, Integrated. The Alcohol Only group showed significant results on frequency of drinking (AFDPS) at 3 months, and on quantity of drinking (AQDPS) at 6 months.

The lower section presents results on two measures of negative consequences. The Combined intervention showed effects on both measures at both assessments. The Alcohol Only and Integrated intervention groups showed effects on both measures at 6 months. The Sex Only group showed effects on sexual consequences (ARSEX) at 6 months.

## 4 Discussion

In this study, the main findings were related to outcomes measuring alcohol-related RSB and outcomes measuring negative consequences. For these outcomes, overall patterns did not differ by intervention content. Most interventions had either remaining effects that could be identified at both follow-ups, or delayed effects identified at the second follow-up. On alcohol outcomes, only short-term effects could be identified and only on quantity of drinking per week. For this outcome, positive effects could be confirmed for all interventions except the one only focusing on alcohol-related RSB.

Results differ somewhat to earlier studies, and also to what was hypothesized in this study. In none of the preceding US studies could positive results be established on negative consequences. In the initial study, effects remained up to 6 months post-intervention, but only remained for one month post-intervention in the second study. In the first study, findings were mainly related to alcohol outcomes. It was emphasized that alcohol PNF only improved alcohol use, while an alcohol-related risky sexual behavior PNF only improved alcohol use in conjunction with sex. Such distinct correlations between intervention content and intervention outcome could not be established in the present study, i.e., PNF specific to drinking in sexual situations was not needed to reduce alcohol-related RSB.

The present findings were mainly related to outcomes on alcohol-related RSB and negative consequences, and the indistinct relationship between intervention content and intervention effect. One explanation could be that participants had explicitly been invited to participate in a sex and alcohol intervention study. This may have caused participants motivated for a change in these areas consenting to participation, and participants may have responded to assessments in a way they felt acceptable (Colagiuri, 2010, Kypri et al., 2015). A second explanation could be that, since alcohol use and alcohol-related RSB are closely related, and since intervention contents partly overlap, the distinct results reported by Lewis and coworkers (2014) are simply difficult to replicate. In general, it is difficult to replicate the same statistical results as those found in preceding studies (Aarts et al., 2015, Patil et al., 2016).

An interesting finding from the present study is the delayed significant effects identified on both outcomes measuring negative consequences. Such effects were not reported in the two US studies, but it seems logical that changes in behavior patterns are followed by corresponding changes in negative consequences. Considering results from the present study and preceding studies, the overall interpretation is that personalized normative feedback interventions have meaningful but varying effects on important outcome variables.

The present study is not without limitations. A major weakness is the 11% response rate at the initial screening, which may have biased results. The possibility of obtaining good response rates by using email is diminishing: In a study that used the same methodology for recruiting students at one of the participating universities four years before this study, a response rate of 34% was achieved (Källoff, Thomasson, Wahlgren, & Andersson, 2015). Another limitation concerns the use of sexual orientation identity as inclusion criteria. Hazardous drinking and alcohol-related RSB occur in all groups and not only in heterosexuals (Hughes, Wilsnack, & Kantor, 2016). The heterosexuality criteria were also used by Lewis and co-workers (2014), and the intervention content has not yet been adapted to other sexual orientation identities. Adapting the content and including all students, regardless of sexual orientation, is an important issue for future studies. Another weakness is that there are no specific inclusion criteria for alcohol-related RSB. In the present study, well-established criteria for hazardous drinking were used, and being sexually active does not necessarily imply alcohol-related RSB. Additionally, the definition of alcohol-related RSB, intervention content on alcohol-related RSB, and outcome measures on alcohol-related RSB, is limited to engaging in sexual behavior with multiple or casual partners under the influence of alcohol. Future studies could focus on other important aspects of alcohol-related RSB, such as sexual assault and victimization (Gilmore, Lewis, & George, 2015).

### 4.1 Conclusion

This replication study confirms that the same personalized normative feedback interventions, previously evaluated in US college students and now applied to Swedish university students, could be useful in reducing alcohol use, alcohol-related RSB, and their negative consequences.

## 5 Role of funding source

This research was supported by grant 03342-2018 from the Swedish National Institute of Public Health awarded to Dr. Claes Andersson. The funding body had no role in the study design, collection, analysis or interpretation of the data, writing the manuscript, or the decision to submit the paper for publication.

## 6 Contributors

The author conducted all parts of the present study.

## Ethical approval

The study was approved by the Regional Ethics vetting board, file numbers 2017/662 and 2017/907.

## CRediT authorship contribution statement

**Claes Andersson:** Conceptualization, Data curation, Formal analysis, Funding acquisition, Investigation, Methodology, Project administration, Resources, Software, Supervision, Validation, Visualization, Writing-original draft, Writing-review & editing.

## Declaration of Competing Interest

The author declare that they have no known competing financial interests or personal relationships that could have appeared to influence the work reported in this paper.
